# MPLA and AddaVax^®^ Adjuvants Fail to Promote Intramuscular LaAg Vaccine Protectiveness against Experimental Cutaneous Leishmaniasis

**DOI:** 10.3390/microorganisms9061272

**Published:** 2021-06-11

**Authors:** Diogo Oliveira-Maciel, Júlio Souza dos-Santos, Gabriel Oliveira-Silva, Mirian França de Mello, Alessandra Marcia da Fonseca-Martins, Monique Pacheco Duarte Carneiro, Tadeu Diniz Ramos, Luan Firmino-Cruz, Daniel Claudio Oliveira Gomes, Bartira Rossi-Bergmann, Herbert Leonel de Matos Guedes

**Affiliations:** 1 Laboratório de Imunofarmacologia, Instituto de Biofísica Carlos Chagas Filho, Universidade Federal do Rio de Janeiro, Rio de Janeiro 21941-902, Brazil; diogo.omac@gmail.com (D.O.-M.); Julioiebeu@gmail.com (J.S.d.-S.); gabriel.oliveira1207@gmail.com (G.O.-S.); alemfmartins@gmail.com (A.M.d.F.-M.); monique@biof.ufrj.br (M.P.D.C.); tadeuramos.10@gmail.com (T.D.R.); luancruz_rj@hotmail.com (L.F.-C.); bartira@biof.ufrj.br (B.R.-B.); 2 Laboratório Interdisciplinar de Pesquisas Médicas, Instituto Oswaldo Cruz, Fundação Oswaldo Cruz, Rio de Janeiro 21040-360, Brazil; 3 Grupo de Imunologia e Vacinologia, Instituto de Microbiologia Paulo de Goés, Universidade Federal do Rio de Janeiro, Rio de Janeiro 21941-902, Brazil; mirianfrancafm@gmail.com; 4 Núcleo de Doenças Infecciosas/Núcleo de Biotecnologia-Universidade Federal do Espírito Santo, Vitória 29075-910, Brazil; DGomes@ndi.ufes.br

**Keywords:** *Leishmania amazonensis*, LaAg vaccine, adjuvants, MPLA, AddaVax^®^, intramuscular, immunization, C57BL/6

## Abstract

There is so far no vaccine approved for human leishmaniasis, mainly because of the lack of appropriate adjuvants. This study aimed to evaluate in mice the capacity of a mixture of monophosphoryl lipid A (MPLA) and AddaVax^®^ adjuvants in enhancing the efficacy of a Leishvacin^®^-like vaccine comprised of *Leishmania amazonensis* whole antigens (LaAg). For that, mice were immunized with LaAg plus MPLA/AddaVax^®^ by the intramuscular route (i.m.) prior to challenge with 2 × 10^5^ and 2 × 10^6^ living parasites. Immunization with LaAg alone reduced the lesion growth of the 2 × 10^5^-challenged mice only in the peak of infection, but that was not accompanied by reduced parasite load, and thus not considered protective. Mice given a 2 × 10^6^ -challenge were not protected by LaAg. The association of LaAg with MPLA/AddaVax^®^ was able to enhance the cutaneous hypersensitivity response compared with LaAg alone. Despite this, there was no difference in proliferative cell response to antigen ex vivo. Moreover, regardless of the parasite challenge, association of LaAg with MPL/AddaVax^®^ did not significantly enhance protection in comparison with LaAg alone. This work demonstrated that MPL/AddaVax^®^ is not effective in improving the efficacy of i.m. LaAg vaccine against cutaneous leishmaniasis.

## 1. Introduction

Leishmaniasis is a set of diseases caused by the infection of several species of the protozoan parasite of the genus *Leishmania*, being endemic in approximately 98 countries [1]. *Leishmania amazonensis, L. braziliensis, L. guyanensis*, *L. tropical*, *L. aethiopica*, *L. martiniquensis*, *L. orientalis,* and *L. major* are the species that typically cause cutaneous leishmaniasis, with *L. amazonensis* being the species that can cause an anergic form of the disease [2,3,4]. Although there is a treatment for leishmaniasis, it is noted that the drugs used trigger high toxicity; besides this, parasite resistance has been described in the Indian subcontinent and South America [5], as well as in the Mediterranean area [6], indicating the need for alternative measures to fight the disease. In view of the variability of the *Leishmania* species, adapted to different ecosystems, different species of the sandfly vector, and the parasitism of different animal species [7], this means that efforts to control vectors and reservoirs have limited efficiency. When exposed to the live parasite, the host immune system is capable of recognizing the pathogen and, in the face of this challenge, triggers immune responses in order to kill the parasite. In addition, there is a generation of memory cells that are able to induce a quick and efficient response upon re-infection [8]. Thus, the strategy of using first generation vaccines (dead, attenuated parasites or total antigens) is used in order to build an adaptive immune response that resembles the response that would naturally occur during an infectious process [9]. Another strategy to highlight is leishmanization, a technique licensed for use in Middle Eastern countries for high-risk populations, which is configured by a mixture of *L. major* parasites, both alive and dead [10]. This technique proves to be efficient against Old World leishmaniasis; however, there are inherent difficulties in the standardization of *Leishmania* used in the inoculum [9].

Previous work has described that a vaccine composed of five dead *Leishmania* promastigote strains could induce protective immunity to cutaneous leishmaniasis in humans and in experimental models [11]. However, one of the problems arising from this vaccine would be in the control of the complexity of its composition and the need to incorporate different strains of *Leishmania*. Then, Mayrink et al. [12] carried out a study comparing the immunogenic profile of the antigenic extracts of each of the five species that make up the vaccine in C57BL/10 mice. It was shown that Leishvacin^®^ composed of antigens of the five strains ((*L. (L.) amazonensis* (IFLA/BR/67/PH8); *L. (L.) mexicana* (MHOM/BR/60/BH06); *L. (Viannia) guyanensis* (MHOM/BR/70/M1176); *L. (L) major*-like [(MHOM/BR/71/BH49), *L. (L) major*-like [(MHOM/BR/73/BH121)), as well as the PH8 monovalent vaccine, induced protective immunity in mice infected with *L. amazonensis* [12]. Furthermore, the stimulus generated by immunization with Leishvacin^®^ triggers a partially protective response [13], with an increase in IFN-γ levels in BALB/c mice. However, human protection in clinical trials using adjuvant-free Leishvacin^®^ did not prove effective and the manufactured Leishvacin^®^ was thus discontinued [14]. It has already been demonstrated that, by the intramuscular route, immunization with LaAg was able to induce a counterprotective effect in BALB/c mice [15]. When evaluated in C57BL/6 challenged with 2 × 10^6^ parasites, LaAg alone did not induce protection.

Immunological adjuvants are substances that have the purpose of promoting or enhancing the immunogenicity of antigens present in the vaccine formulation, as several antigens present in a vaccine are not very immunogenic [16]. Adjuvants may be used to improve and rescue old vaccines like Leishvacin^®^. When LaAg was in combination with *Cryptosporidium parvum* [17], IL-12 [18], or Bacillus Calmette-Guérin (BCG) [19,20], partial protection was reported. Furthermore, new adjuvants, such as monophosphoryl lipid A (MPLA) and AddaVax^®^, have since been used in vaccine development and clinical trials. MPLA associated with three antigens (glycoprotein 63; cysteine proteinases; and a membrane-bound acid phosphatase, Leish-F1 vaccine) was first tested in a murine model of cutaneous leishmaniasis caused by *L. mexicana* [21]. AddaVax^®^ is an oil-in-water nanoemulsion based on squalene (SE), which has a formulation similar to the adjuvant MF59, which is licensed in Europe to constitute the flu vaccine [22]. MF59 is known to be capable of triggering cellular immune responses through its deposit effect [23,24].

Several studies conducted by the Infectious Disease Research Institute (IDRI), located in Seattle, United States, found that MPLA associated to an oil–water formulation (MPLA-SE) conferred an increase in effectiveness against leishmaniasis [25,26,27]. In 2002, Leish-111f was associated to MPLA-SE and enhanced the control of lesion size in experimental models of leishmaniasis caused by *L. major* and *L. amazonensis*. In 2007, the same vaccine induced protection through the control of parasite load in the spleen of an *L. infantum* experimental model. The inclusion of MPLA-SE has also been demonstrated to induce a strong Th1-type immune response, which had high levels of IFN-γ and comparatively low levels of IL-4 and IL-10 [25,26,27]. When analyzing the serum immunoglobulin levels, a higher IgG2a/IgG1 ratio was observed when MPLA was present in the vaccine formulation [25]. Using the same constituents of MPLA-SE, to maintain the same properties of the adjuvant, this work evaluated MPLA/AddaVax^®^. In order to investigate the use of MPLA/AddaVax^®^ as an adjuvant in the LaAg vaccine administered by the intramuscular route, experiments were performed using two different parasite doses as the challenge.

## 2. Materials and Methods

### 2.1. Animals

Female C57BL/6 and BALB/c mice, 6–8 weeks old (*n* = 5 per group), were obtained from the breeding facility of UFRJ. All animals were kept in mini-isolators (Alesco, São Paulo, Brazil) and kept under controlled temperature and light/dark cycles of 12 h/12 h, in addition to receiving filtered water and commercial feed (Nuvilab, Curitiba, Paraná, Brazil). The experiments were carried out in accordance with the Ethics Committee on the Use of Animals of the Health Sciences Center of the Federal University of Rio de Janeiro (Comitê de Ética no Uso de Animais do Centro de Ciências da Saúde da Universidade Federal do Rio de Janeiro), under the protocol number: IBCCF 157).

### 2.2. Parasites

*Leishmania amazonensis* (MHOM/BR/75/Josefa) was purified from the lesions of infected BALB/c mice and maintained in M199 medium (Sigma-Aldrich, San Luiz, MI, USA) supplemented with 0.02% hemin, 10% heat-inactivated fetal bovine serum (FBS) (Cultilab, Campinas, São Paulo, Brazil), 100 U/L penicillin, and 100 μg/L streptomycin at 26 °C prior to the in vivo experimental infections. For in vivo infection, promastigotes in the stationary phase of growth were used, promastigotes were washed three times in phosphate buffered saline (PBS) at 800× g for 10 min, and the pellet was resuspended in PBS. To ensure infectivity, the parasites were only used in experiments up to the fifth passage in in vitro culture.

### 2.3. Immunization by Intramuscular Route

Mice were anesthetized by inhalation using isoflurane (Cristália, Fortaleza, Ceará, Brazil). Using a 1 mL 29 G syringe (BD, Franklin Lakes, NJ, USA), immunization with total antigen of *L. amazonensis* (LaAg), prepared as previously described [28], was performed in the posterior muscular region of the right hind footpad so that the syringe, when introduced, maintained an angle of 45°. After one week, the animals received a booster dose at the same dosage (Supplementary Materials). Control animals received only the same volume of PBS. Administration was as follows: 100 µg of LaAg per dose (20 µL of a 5 mg/mL solution), 20 µg of MPLA (5 µL), and 50% of the final volume (50 µL) was AddaVax^®^ (Invivogen, San Diego, CA, USA). Moreover, PBS was used to make up the final volume of 100 µL.

### 2.4. Infection, Measurement of Lesions and Hypersensitivity

C57BL/6 mice were infected subcutaneously in the right hind footpad using a syringe (HAMILTON, Reno, NV, USA) with 2 × 10^5^ and 2 × 10^6^ stationary-phase promastigotes of *L. amazonensis* in 20 µl of PBS.

After infection, the vertical lesion thickness was monitored weekly by pachymetry using a caliper (*Mitutoyo*, Takatsu-ku, Kawasaki, Kanagawa, Japan). To assess hypersensitivity, the footpad size was also assessed 18 h, 24 h, and 48 h after infection (Supplementary Materials).

### 2.5. Parasite Load by Limiting Dilution Assay (LDA)

After euthanizing the mice, the infected footpads were removed and placed in 70% alcohol for 1 min for disinfection. The footpads were then macerated using a tissue mixer with 1 mL M199 medium (Sigma-Aldrich, St. Louis, MI, USA) supplemented with 0.02% hemin, 10% FBS (Cultilab, Campinas, São Paulo, Brazil), 100 U/L penicillin, and 100 μg/L streptomycin. Then, a 96-well plate was pre-filled with 150 μL M199 medium supplemented as above and 50 μL of the macerated cell suspension was placed in the first well. A 1:4 dilution series was performed by passing 50 μL of the dilution to the subsequent well for a total of 24 dilutions for each sample. The plates were incubated in a bio-oxygen demand (BOD, Erie, Pittsburgh, PA, USA) incubator at 26 °C for 14 days. The presence of promastigotes was examined on an optical microscope (Olympus, Shinjuku, Toquio, Japan), and the last well containing promastigotes was recorded in order to calculate the parasite load. The calculation used to determine the parasite load was as follows: number of parasites = 4*^x^* ÷ (mass of organ in grams), where *x* is the number of the last well in which parasites were observed. Effective protection was considered when parasite load was controlled.

### 2.6. Lymphoproliferation

The lesions were removed at 72 h after infection with 2 × 10^5^ *L. amazonensis* and single-cell suspensions were prepared (Supplementary Materials). The cells were plated in a 96-well plate at a concentration of 1 × 10^6^ cells/mL and were stimulated with 10 µg/mL of *L. major* antigen (LmAg) for 72 h at 37 °C with 4% CO_2_. Cell viability was assessed by the MTT [3-(4,5dimethylthiazol-2-yl)-2,5-diphenyltetrazolium bromide] assay, in which 20 μL of a 5 mg/mL MTT dye solution (Sigma-Aldrich, St. Louis, MI, USA) was added to each well and incubated for 4 h. After incubation, 100 μL SDS–HCl (10% SDS in 0.01 N HCl) in each well was added to dissolve the MTT formazan produced. The relative amount of formazan produced by viable cells per well was measured photometrically at 570 nm using a plate reader (Spectra Max M5, San Jose, CA, USA).

### 2.7. Flow Cytometry

Popliteal lymph nodes were removed and macerated as described earlier. Briefly, cells were quantified by light microscope using trypan blue and then plated at 1 × 10^6^ cells/well in a 96-well plate. Staining of intracellular and extracellular markers was performed following the manufacturer’s instructions. The cells were re-stimulated ex vivo for 4 h with phorbol 12-myristate 13-acetate (PMA; 20 ng/mL) plus ionomycin (1 μg/mL) in the presence of a Golgi complex inhibitor (brefeldinA, St. Louis, MI, USA) for intracellular cytokine analysis. Extracellular markers were stained, and the cells were fixed and permeabilized to enable intracellular staining. The antibodies used in this work were as follows: anti-CD3-Pacific Blue (eBioscience, San Diego, CA, USA) (1:200), anti-CD4-PECy7 (eBioscience, San Diego, California, USA) (1:200), and anti-CD8-PercP (eBioscience, San Diego, CA, USA) (1:200) for the extracellular markers, and IFN-γ-APC (eBiosciences, San Diego, CA, USA) (1:100) for the intracellular marker. Analysis was performed in the FlowJo software (Ashland, OR, USA).

### 2.8. Statistics

The bar graphs were analyzed by Student’s t-test and the XY graphs, such as those of the lesion development, were assessed by two-way analysis of variance (ANOVA) using Bonferroni’s post-test. Statistical analysis was performed using the GraphPad Prism v.5 software and the differences were considered significant when *p* ≤ 0.05.

## 3. Results

### 3.1. Intramuscular LaAg Vaccine Induces Partial Protection in the Lesion Size of C57BL/6 Mice Infected with 2 × 10^5^ Promastigotes, But Not with 2 × 10^6^ Promastigotes

**Figure 1:**
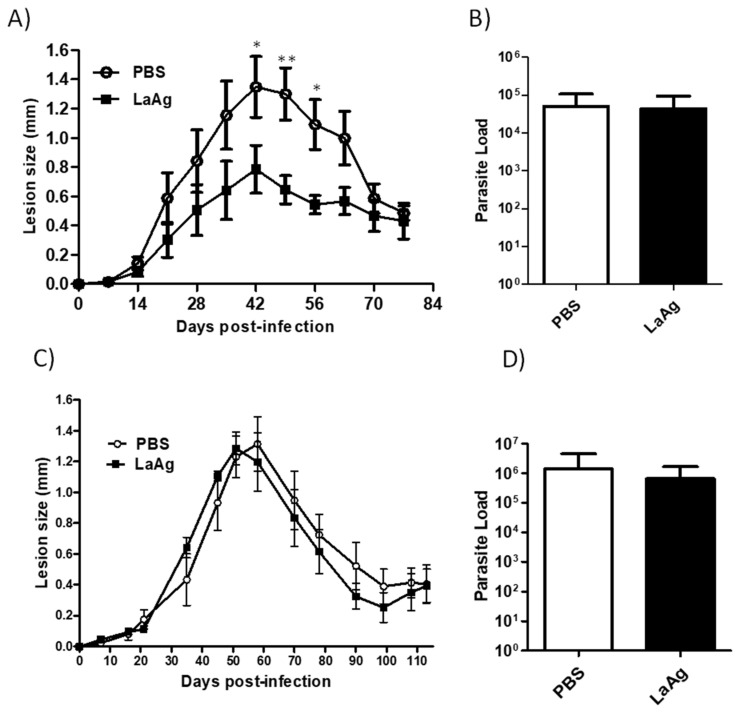
C57BL/6 mice immunized with the intramuscular LaAg vaccine showed partial reduction in the lesion size when infected with 2 × 10^5^ promastigotes. Mice were injected with the LaAg vaccine or phosphate buffered saline (PBS) and then infected with 2 × 10^5^ (**A**,**B**) and 2 × 10^6^ (**C**,**D**) *L. amazonensis* stationary phase promastigotes in the right hind footpad. The lesion development was monitored using a caliper on the indicated days until the 77th (**A**) day post-infection and 113th (**C**) day post-infection. (**B**,**D**) The footpads were removed at the end of the respective periods, macerated and used in an limiting dilution assay (LDA) to determine the parasite load. Data are representative of two independent experiments (mean ± standard deviation; *n* = 5). ** *p* < 0.01, * *p* < 0.05; assessed by two-way analysis of variance (ANOVA) using Bonferroni’s post-test.

Intramuscular administration of the LaAg vaccine has previously been demonstrated to increase the susceptibility of BALB/c mice to *L. amazonensis* [15], but no effect was observed in C57BL/6 mice when challenged with 2 × 10^6^ promastigotes. In the present study, the LaAg vaccine was administered intramuscularly in C57BL/6 mice and then challenged with two different doses of *L. amazonensis*, 2 × 10^5^ and 2 × 10^6^ promastigotes. The control group (injected with PBS and then infected) and mice immunized and then infected with 2 × 10^6^ presented the same lesion size and parasite load profile (Figure 1C,D), similar to that observed before. On the other hand, despite the fact that there was no difference in parasite load, mice immunized and then infected with the smaller parasite inoculum had reduced lesion sizes from days 35 to 63 post-infection in comparison with the control group (Figure 1A,B). This result shows that the intramuscular LaAg vaccine is partially effective in controlling the lesion size with a challenge of 2 × 10^5^ promastigotes, but does not control the parasite load in C57BL/6.

### 3.2. Intramuscular LaAg Vaccine Induces a Strong Delayed Hypersensitivity Response When Associated with the Adjuvants MPLA/AddaVax^®^

**Figure 2:**
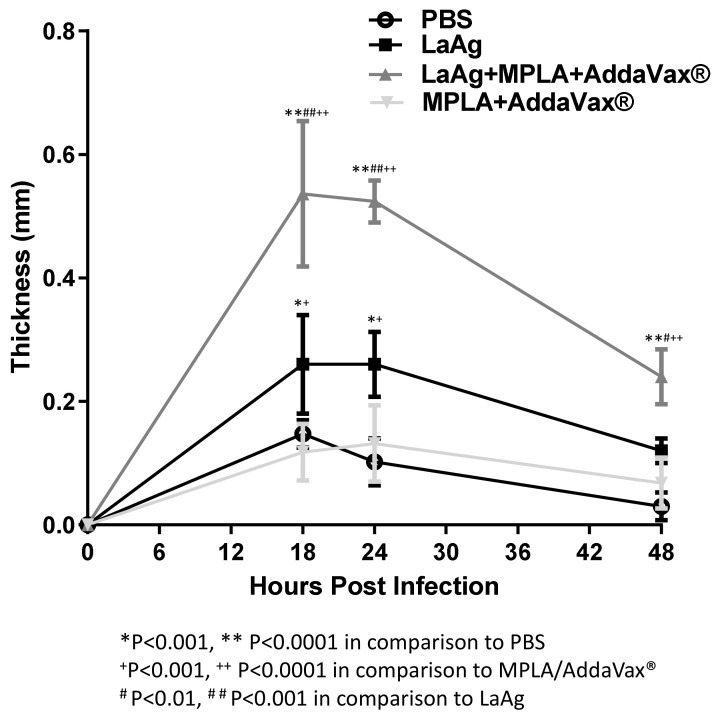
Intramuscular LaAg vaccine associated with the adjuvants monophosphoryl lipid A (MPLA)/AddaVax^®^ induces a strong delayed hypersensitivity response. Mice were immunized with LaAg and MPLA/AddaVax^®^ alone or together, while controls received PBS alone. Animals were then challenged with 2 × 10^5^ *L. amazonensis* stationary phase promastigotes in the right hind footpad and the kinetics of the hypersensitivity response were scored at 18, 24, and 48 h post-infection. Data are representative of two independent experiments (mean ± standard deviation; *n* = 5) ** *p* < 0.0001, * *p* < 001 in comparison with PBS; ^+^ *p* < 0.001, ^++^ *p* < 0.0001 in comparison with MPLA/AddaVax^®^; ^#^ *p* < 0.01, ^##^ *p* < 0.001 in comparison with LaAg; assessed by two-way ANOVA using Bonferroni’s post-test.

To investigate the effect of immunization using LaAg alone or associated with the adjuvants, MPLA/AddaVax^®^, in the delayed-type hypersensitivity (DTH) response in C57BL/6 mice, *L. amazonensis* infection was used as challenge and the cellular response was assessed by pachymetry. Similar to that observed in BALB/c [29], C57BL/6 immunized with LaAg induced hypersensitivity in comparison with the PBS-injected control group at 18, 24, and 48 h after infection (Figure 2). In an attempt to enhance the immunogenicity of the LaAg vaccine, the adjuvants MPLA and AddaVax^®^ were added. MPLA/AddaVax^®^ alone did not induce DTH in mice that received this formulation in comparison with the control group (PBS) over the 48 h period or compared with the mice that received the LaAg vaccine alone at 18 and 24 h. However, the association of intramuscular LaAg vaccine with MPLA/AddaVax^®^ was capable of inducing a stronger DTH response than all other groups over the 48 h period.

### 3.3. Immunization with LaAg Alone or LaAg and MPLA/AddaVax^®^ Induces Proliferation of Immune Cells

**Figure 3:**
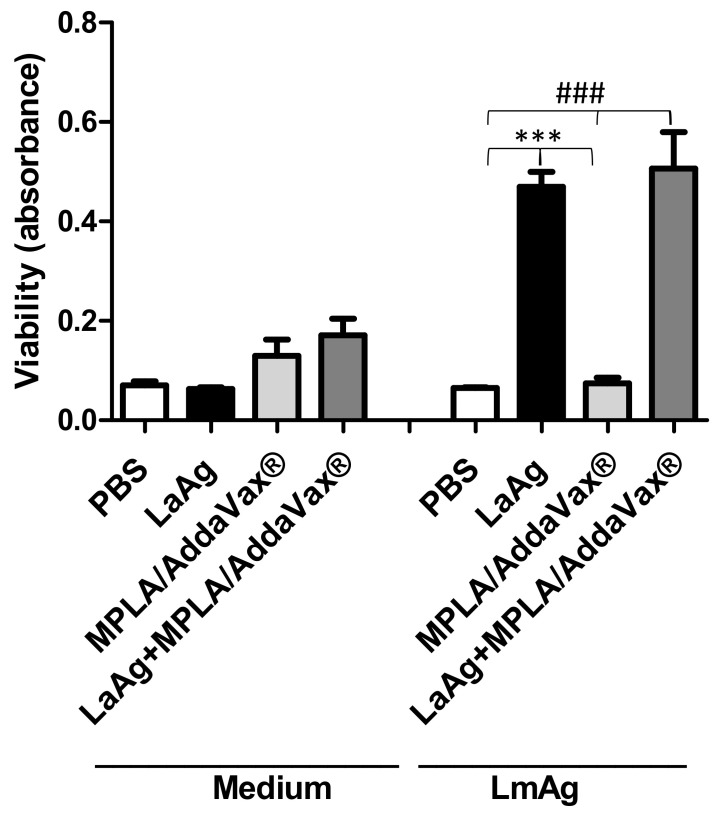
Immunization of LaAg with MPLA plus AddaVax^®^ induced proliferation of immune cells similar to those immunized with LaAg alone. Cells from the lymph node draining of the lesion of C57BL/6 mice immunized or PBS-injected were removed 72 h after infection with 2 × 10^5^ *L. amazonensis* stationary phase promastigotes. Single cell suspensions (1 × 10^6^) were stimulated with 10 μg/mL *L. major* antigen (LmAg) or medium as a control. Data are representative of two independent experiments (mean ± standard deviation; *n* = 5) *** *p* < 0.0001 in relation to cells from the LaAg-immunized group stimulated with LmAg and ^###^ *p* < 0.0001 in relation to cells from the LaAg+MPL/Addavax^®^-immunized group stimulated with LmAg.

To evaluate the T cell response in immunized mice, an ex vivo cell lymphoproliferation assay was performed. There were no differences between groups that were not stimulated with *L. major* antigen (LmAg) ex vivo (Figure 3). However, mice immunized with LaAg were capable of proliferation upon stimulation with interspecific antigen in comparison with the control group (PBS) and mice that received MPLA/AddaVax^®^ only. Furthermore, similar proliferation was observed for the cells from mice immunized with the LaAg vaccine with MPLA/AddaVax^®^.

### 3.4. LaAg Vaccine Associated with MPLA/AddaVax^®^ Displays the Same Profile As the LaAg Vaccine in C57BL/6 Mice

**Figure 4:**
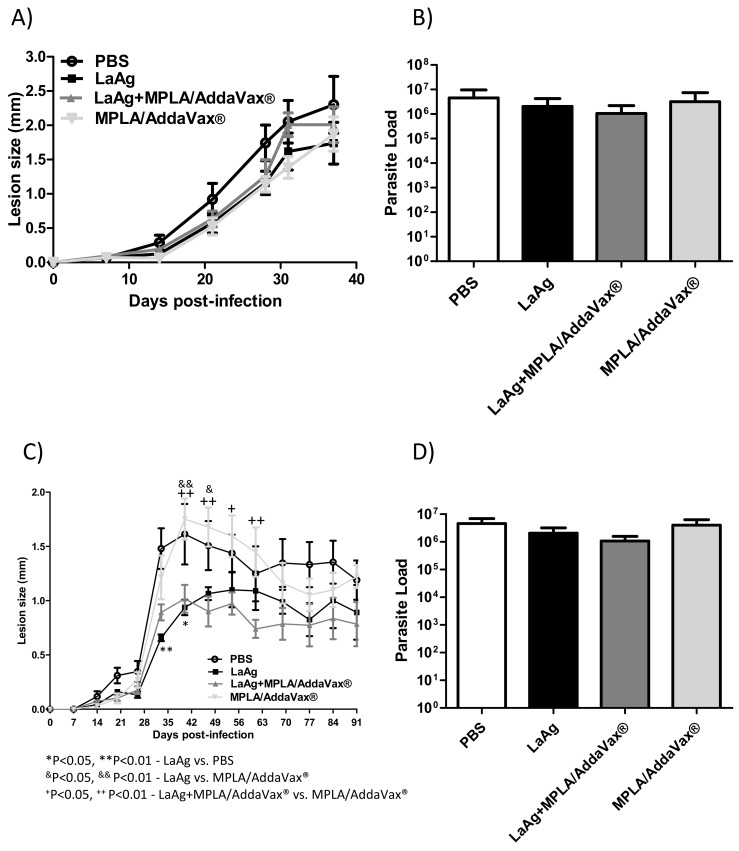
The combination of intramuscular LaAg vaccine and MPLA/AddaVax^®^ failed to enhance the protection in C57BL/6 mice infected with *L. amazonensis*. Mice were immunized with LaAg alone, LaAg + MPLA/AddaVax^®^, or MPLA/AddaVax^®^ alone and, a week later, the mice received a booster dose. After a further 7 days, mice were challenged with 2 × 10^5^ or 2 × 10^6^ *L. amazonensis* stationary phase promastigotes in the right hind footpad. The lesion development was monitored using a caliper on the indicated days until the 37th (2 × 10^5^) (**A**) and 91st (2 × 10^6^) (**C**) day post-infection. (**B**,**D**) The footpads were removed at the end of the respective experiments, macerated and used in an LDA to determine the parasite load. Data are representative of two independent experiments (mean ± standard deviation; *n* = 5). * *p* < 0.05, ** *p* < 0.01-LaAg vs. PBS, ^&^*p* < 0.05, ^&&^ *p* < 0.01-LaAg vs. MPLA/AddaVax^®^; ^+^ *p* < 0.05, ^++^ *p*<0.01-LaAg + MPLA/AddaVax^®^ vs. MPLA/AddaVax^®^; assessed by two-way ANOVA using Bonferroni’s post-test.

To investigate the impact of the combination of the intramuscular LaAg vaccine with MPLA/AddaVax^®^ on *L. amazonensis* infection in vivo, the animals were infected with two doses of promastigotes, 2 × 10^5^ and 2 × 10^6^. Using 2 × 10^6^ promastigotes, LaAg with MPLA/AddaVax^®^ did not induce any protection and was similar to the profile of mice immunized with the LaAg vaccine alone until 37 days post-infection (Figure 4A), with no effect on parasite burden (Figure 4B). Using 2 × 10^5^ promastigotes, LaAg with MPLA/AddaVax^®^ presented the similar partial protection in the lesion size as that of LaAg-immunized mice, without affecting the parasite load (Figure 4C,D). Furthermore, the mice that received LaAg with MPLA/AddaVax^®^ presented a lower lesion size in comparison with mice that received MPLA/AddaVax^®^ alone or PBS from day 40 until 61 post-infection in those challenged with 2 × 10^5^ promastigotes. Meanwhile, mice that received LaAg alone only presented a lower lesion size between days 33 and 40 compared with the PBS control group and between days 40 and 47 in mice that received MPLA/AddaVax^®^.

Immunization with LaAg and MPLA/AddaVax^®^ is not able to induce CD4^+^ and CD8^+^ IFN-γ^+^ T cells.

**Figure 5:**
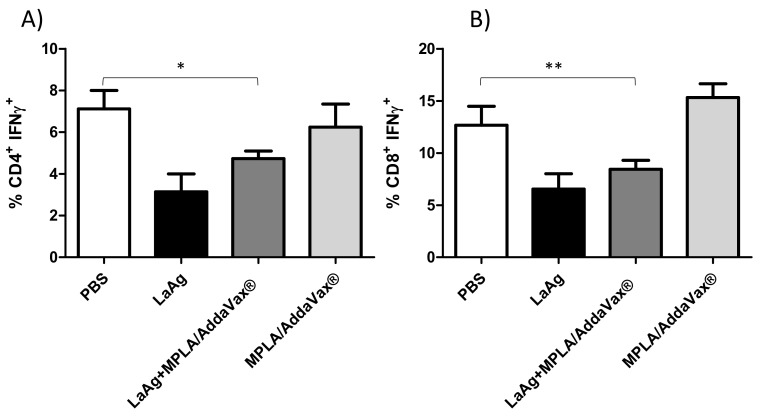
Profile of intracytoplasmic cytokines of CD4^+^ and CD8^+^ IFN-γ^+^ T cells. Lymph node cells from mice infected with 2 × 10^5^ *L. amazonensis* promastigotes for 77th days were plated (1 × 10^6^ per well) and restimulated for 4 h with phorbol 12-myristate 13-acetate (PMA) (20 ng/mL) and ionomycin (1 µg/mL), and then stained for flow cytometry analysis. After a previous gate (CD3^+^CD4^+^) and (CD3^+^CD8^+^), the percentages of CD4^+^ IFN-γ^+^ T cells (**A**) and CD8^+^ IFN-γ^+^ T cells (**B**) were determined. The data are representative of one independent experiment (mean ± standard deviation; *n* = 5) * *p* <0.05- CD4^+^ IFN-γ^+^ T cells, ** *p* < 0.01-CD8^+^ IFN-γ^+^ T cells, obtained from Student’s t-test.

To evaluate the profile of intracytoplasmic cytokines of T cells triggered in immunized mice, an ex vivo flow cytometry assay was performed. Mice that received LaAg or LaAg with MPLA/AddaVax^®^ were not able to induce proliferation of CD4^+^ and CD8^+^ IFN-γ^+^ T cells in relation to the PBS control group and those that received only adjuvants (MPLA/AddaVax^®^). This assay demonstrated that immunization with MPLA and AddaVax^®^ adjuvants when associated with LaAg was unable to induce cytokine production by these cells (Figure 5).

## 4. Discussion

Immunization is a technique of great applicability in the eradication and prevention of diseases caused by different pathogens [30]. The goal of immunization is to generate a strong immune response to the administered antigen, which is capable of providing long-term protection so that the immunized individuals do not manifest illnesses when naturally exposed to the pathogens [8]. With the ever-increasing need to combat diseases, the effectiveness of vaccines in controlling diseases has greatly impacted both human and animal health throughout history, providing an adequate environment for the development of vaccines on commercial scales [30]. The use of mouse models is a fundamental step in the evaluation of vaccine candidates and to establish the protocols to better assess vaccine protection [31]. The choice of mouse model is an important criterion, which can affect the efficacy of a tested vaccine owing to the intrinsic genetic differences between mouse strains.

In an *L. amazonensis* infection, Pinto et al. [32] compared the profile of infection by the intradermal route in C57BL/6 mice with two inoculums of 10^3^ and 10^6^ of the PH8 strain. Mice infected with the higher inoculum developed larger lesions approximately 9 weeks after infection; however, after this time, the lesions were equal until the end of the experiment at 22 weeks of infection. The same profile was observed in relation to the parasitic load. However, it is worth mentioning that, at the peak phase of infection until the end of the experiment, the number of parasites was similar. Recently, dos-Santos et al. [33] reported that, during infection with the *L. amazonensis* Josefa strain in Sv129 mice, although there was a difference in the time of lesion development between inoculums of 2 × 10^5^ and 2 × 10^6^, the lesions were equal at the end of 16 weeks of infection and there was not a difference in the parasite load at the end. Thus, the inoculation dose can greatly influence the profile of *Leishmania* infection.

Here, the evaluation of the vaccine in the model was challenged by infection using two different inoculation doses. The first was an infection with a smaller number of parasites by inoculation with 2 × 10^5^ *L. amazonensis* promastigotes in comparison with the second, where a tenfold increase in the number of parasites was used in the challenge (2 × 10^6^). In this context, faced with a 2 × 10^6^ challenge, it was not possible to observe the control of either the lesion size or the parasite load at the inoculation site in relation to PBS and LaAg vaccine groups, which is in accordance with the work of Pinheiro et al. [15]. With the 2 × 10^6^ challenge, the LaAg vaccine by the intramuscular route worsened the lesion size and parasite load in comparison with the PBS group [34]. However, different from that observed when the LaAg vaccine was administered by the intranasal route, the LaAg vaccine was capable of protection in terms of both lesion size and parasite load, when challenged with 2 × 10^6^ parasites [28]. This shows that the LaAg vaccine is effective by the intranasal route even in a scenario with a higher parasite inoculum.

The use of adjuvants serves several purposes, such as enhancing the immunogenic properties of individuals by reducing the number of doses to provide protection; reducing the amount of antigens needed to achieve a protective state; increasing the duration of the induced immune response (cytotoxic or mucosal response); expanding epitope recognition to ensure greater coverage of the immune response; and activating the immune response in poor responders, as in the case of neonates and the elderly [35,36]. Despite a lot of controversy, it is known that, in murine models, the Th1 response is essential for control/protection and self-resolution in *L. amazonensis* infection, while the Th2 response is associated with the susceptibility/progression of the disease. The stimulus generated by immunization with Leishvacin^®^ can trigger a partially protective response in humans, inducing a Th1 response [37]. Leishvacin^®^ has been tested once in Colombia and Ecuador in phase 3 clinical trials, and in general, it was found to be safe and immunogenic, but actually did not provide protection against *L. panamensis* infection [14]. A possible explanation is related to the fact that the causative agents of the disease are species other than *L. amazonensis.*

In general, the adjuvant association aims to enhance the vaccine following the previously mentioned characteristics. As an example, the Leish-F1 vaccine, which, in clinical trials, was shown to be effective against cutaneous and mucosal leishmaniasis, both in mice and in non-human primates. The association of MPLA-SE (the same constituent of MPLA/AddaVax^®^) in the vaccine has been shown to be safe and immunogenic in healthy humans during phase I and II clinical trials [31]. In immunized and uninfected mice, this triggers an increase in the production of IFN-γ, IL-2, and TNF-α by T CD4^+^ cells, potentiating a Th1 response. In the present study, the association of MPLA/AddaVax^®^ adjuvants did not induce an effect greater than the LaAg vaccine alone, thus the use of these adjuvants is not satisfactory for the combination with LaAg, differing from the results observed by Coler et al. [38], who demonstrated that the association of these adjuvants was able to potentiate the Th1 response, promoted by the Leish-111f antigen. When Leishvacin^®^ was evaluated in C57BL/6 mice, *C. parvum* was used as adjuvant and a challenge using 10^5^ *L. amazonensis* mice demonstrated a partial protection, but Leishvacin^®^ without *C. parvum* was not evaluated to compare the capacity of protection [17]. These data demonstrate that, although we do not observe a satisfactory effect on the combination of LaAg with adjuvants MPLA/AddaVax^®^, it is important to know that the combination using other adjuvants, for example, *C. parvum*, is able to enhance the immunogenic effect of Leishvacin^®^. Therefore, Leishvacin^®^ has potential, but it needs a safe adjuvant. The immunization with LaAg or LaAg plus MPLA/AddaVax^®^ reduced CD4^+^ and CD8^+^ T cells producing IFN-γ in the draining lymph nodes.

Delayed hypersensitivity is a response characterized by a strong induction of T cells between 24 and 72 h after challenge. The classic DTH that is observed in BCG immunization can be observed after intradermal injection of a *Mycobacterium tuberculosis* antigen preparation. If the host has been previously exposed to the bacteria, swelling and hardening will occur. High thickness is the trademark of DTH [39]. However, in mice, there is the Jones–Mote reaction test, which is characterized by a marked infiltration of basophils, reaching a peak between 18 and 24 h, and disappearing or being absent by 48 h [40]. In humans, DTH was used as diagnostic to evaluate the cellular response of infected or immunized individuals, through use of the Montenegro skin test (MST), one of the markers of the cellular immune response, presenting a sensitivity rate of 94% to 98% [41]. As *Leishmania* parasites are killed by IFN-γ-activated macrophages and are not neutralized by antibodies, individuals with DTH have few parasites in their lesions, while those with only a humoral response are unable to control the parasitic burden [42,43]. In addition, patients with no T cell response exhibit diffuse cutaneous leishmaniasis, a manifestation that can be caused by *L. amazonensis* in Brazil [2].

In mice, the DTH response was correlated with protection against *L. major* infection in BALB/c mice that had recovered from an infection, while the Jones–Mote reaction was associated with an inability to fight infection after immunization by the subcutaneous and intradermal route with *L. major* promastigotes killed by formaldehyde. To evaluate the Jones–Mote response in immunized C57BL/6 mice in this study, infection was used as a challenge and a cellular response was observed. It was noted that LaAg-immunized mice were capable of inducing the Jones–Mote response, reaching a peak between 18 and 24 h, and disappearing or being absent at 48 h in comparison with control group (PBS) and mice that received MPLA/AddaVax^®^ only, and a high Jones–Mote response was observed from mice immunized with the combination of LaAg and MPLA/AddaVax^®^ at the same times observed. According to our previous work, by Guedes et al. [34], LaAg-immunized BALB/c mice induced a Jones–Mote reaction, which decayed after 18 h to 48 h. Vélez et al. [44] assessed the Leish-F1 vaccine associated with MPLA, which was able to induce DTH similarly to that observed in this study, although different time points were evaluated. Thus, although MPLA/AddaVax^®^ was able to enhance the Jones–Mote response in LaAg-immunizated C57BL/6 mice, this did not increase protection.

The cellular response was then assessed through lymphoproliferation. Coler et al. [26] demonstrated that cells from mice that had received a vaccine formulation of Leish-111f plus MPLA/AddaVax^®^ proliferate more than mice that received a formulation of soluble *Leishmania* antigens (SLA) plus recombinant murine IL-12. In this study, similar proliferation of cells from the draining lymph node of mice immunized with LaAg or LaAg and MPLA/AddaVax^®^ indicates that the use of adjuvant did not enhance cell proliferation. Antigens from *L. major* were used to determine the cross-response, as LaAg stimulation induces apoptosis in vitro [45]. These data suggest that, although there was an increase in the DTH, there was no increase in lymphoproliferation, meaning that the addition of the adjuvants did not provide more protection, as it did not generate more specific lymphocytes.

Recently, it was demonstrated that antigen similar to LaAg associated to poly I:C induced partial protection in BALB/c mice, and without adjuvant enhanced the lesion size and parasite load, as expected [46]. Poly I:C is a TLR3 ligand and a possible candidate in vaccine formulation against *Leishmania* spp. However, in the partially resistant mice like C57BL6, it is more difficult to get protection; in this way, future experiments in this model should be performed for better evaluation of the effect of the adjuvant.

According to these data, in the face of a challenge of 2 × 10^5^ or 2 × 10^6^ parasites, the vaccine formulation of LaAg associated with MPLA and AddaVax^®^ presented a lesion profile similar to when the LaAg vaccine is used alone, without exhibiting any control of the parasitic load. These results together with our previous work [15,34] lead us to suggest that the use of the LaAg vaccine is more effective when administered by the intranasal route compared with the intramuscular route investigated here.

## 5. Conclusions

We conclude that the combination of MPLA/AddaVax^®^ adjuvants administered with LaAg through the intramuscular route, although augmenting the hypersensitivity response, fails to induce protection against infection with *L. amazonensis*.

## Data Availability

Data are available on request from the corresponding author with reasonable reason.

## Supplementary Materials

The following are available online at https://www.mdpi.com/article/10.3390/microorganisms9061272/s1, Figure S1. Immunization and challenge protocol. Mice were immunized twice via the intramuscular route in the posterior muscular region of the right hind footpad at intervals of 7 days between each dose (100 μL of the vaccine formulation as described in the Material and Methods) and 7 days after the second immunization the mice were challenged with 2 × 10^5^ or 2 × 10^6^ *L. amazonensis* stationary-phase promastigotes in the right hind footpad and the hypersensitivity and lymphoproliferation assay was performed. The lesion development was monitored by pachymetry during the experiment. At the end of the experiments, the parasite load (LDA) and flow cytometry were evaluated.
