# Supplementary material for: MPLA and AddaVax® Adjuvants Fail to Promote Intramuscular LaAg Vaccine Protectiveness against Experimental Cutaneous Leishmaniasis
Source: Microorganisms. 2021 Jun 11;9(6):1272. doi: 10.3390/microorganisms9061272 (PMC8230739; doi:10.3390/microorganisms9061272)
Supplement: Supplementary file 1 [file microorganisms-09-01272-s001.zip › Supplementary.pdf]

Supplementary information

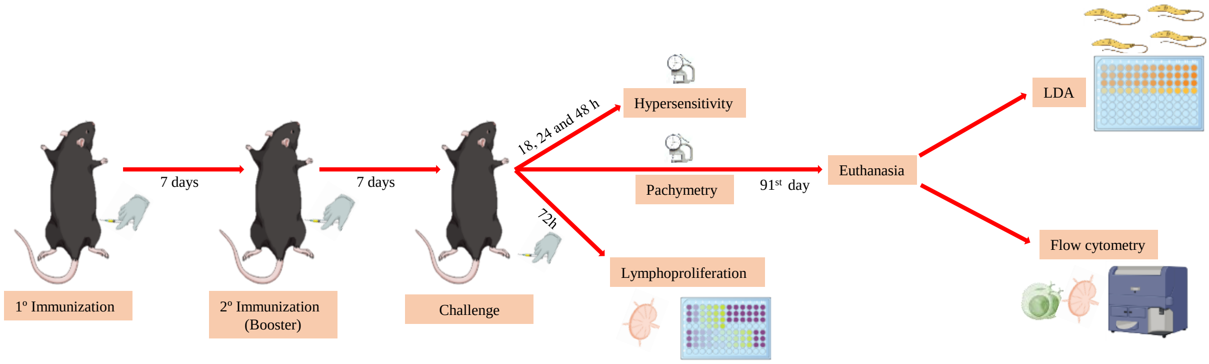

Figure S1. Immunization and challenge protocol. Mice were immunized twice via the intramuscular route in the posterior muscular region of the right hind footpad at intervals of 7 days between each dose (100 µL of the vaccine formulation as described in the Material and Methods) and 7 days after the second immunization the mice were challenged with 2×10^5 or 2×10^6 *L. amazonensis* stationary-phase promastigotes in the right hind footpad and the hypersensitivity and lymphoproliferation assay was performed. The lesion development was monitored by pachymetry during the experiment. At the end of the experiments, the parasite load (LDA) and flow cytometry were evaluated.
